# Genetic characterization of *Toxoplasma gondii* from Qinghai vole, Plateau pika and Tibetan ground-tit on the Qinghai-Tibet Plateau, China

**DOI:** 10.1186/1756-3305-6-291

**Published:** 2013-10-09

**Authors:** Xiao-Xuan Zhang, Zhong-Zi Lou, Si-Yang Huang, Dong-Hui Zhou, Wan-Zhong Jia, Chunlei Su, Xing-Quan Zhu

**Affiliations:** 1 State Key Laboratory of Veterinary Etiological Biology, Key Laboratory of Veterinary Parasitology of Gansu Province, Lanzhou Veterinary Research Institute, Chinese Academy of Agricultural Sciences, Lanzhou, Gansu Province 730046, PR China; 2 Department of Veterinary Medicine, College of Agriculture, Yanbian University, Yanji, Jilin Province 133000, PR China; 3 Department of Microbiology, The University of Tennessee, Knoxville, TN 37996, USA; 4 College of Animal Science and Veterinary Medicine, Heilongjiang Bayi Agricultural University, Daqing, Heilongjiang Province 163319, PR China

**Keywords:** *Toxoplasma gondii*, Genetic typing, PCR-RFLP, Wildlife, Qinghai-Tibet Plateau

## Abstract

**Background:**

The distribution of genetic diversity of *Toxoplasma gondii* in wildlife is of interest to understand the transmission of this parasite in the environment. Limited information on *T. gondii* genotypes has been reported in wildlife in China. The objective of this study was to carry out the genetic characterization of *T. gondii* isolates from wild animals on the Qinghai-Tibet Plateau.

**Methods:**

Using PCR and multilocous polymerase chain reaction-restriction fragment length polymorphism (PCR-RFLP) technology, we detected genetic diversity of *T. gondii* isolates from Qinghai vole, Plateau pika and Tibetan ground-tit in these regions.

**Results:**

In total, 183 brain tissues of different wild animals, including 48 Qinghai vole (*Microtus fuscus*), 101 Plateau pika (*Ochotona curzoniae*) and 34 Tibetan ground-tit (*Pseudopodoces humilis*), were tested for *T. gondii* infection. 11 of these were found to be positive for the *T. gondii* B1 gene by PCR amplification. These positive DNA samples were typed at 10 genetic markers, including 9 nuclear loci (SAG1, 5’-and 3’-SAG2, alternative SAG2, BTUB, GRA6, L358, PK1, c22-8, c29-2), and an apicoplast locus Apico. Six were successfully genotyped at eight or more genetic loci, and were grouped to three distinct genotypes. Four samples belonged to ToxoDB Genotype #10 and the other two samples were identified as two new genotypes (http://toxodb.org/toxo/).

**Conclusions:**

To our knowledge, this is the first report of genetic typing of *T. gondii* isolates in wildlife on the Qinghai-Tibet Plateau, China. The results show that there is a potential risk for the transmission of this parasite through the wildlife in this region.

## Background

The obligate intracellular parasite *Toxoplasma gondii* is an important protozoan that infects warm-blooded vertebrates, including birds and mammals, and one third of the world human population is chronically infected [1-4]. Intermediate hosts such as humans can be infected by ingesting tissue cysts from undercooked meat or consuming food or drinking water contaminated by oocysts shed in the feces of infected cats [5-8].

In general, all *T. gondii* isolates were considered a single species without geographical boundaries, and with limited genetic diversity [5,9]. However, recent studies of *T. gondii* in humans and animals in South America indicated that these isolates are genetically and biologically different from those in North America and Europe where the *T. gondii* population structure is highly clonal and composed mainly of 4 distinct lineages, i.e., Types I, II, III and 12 [5,10-16]. The severe toxoplasmosis in immunocompetent human patients was associated with atypical genotypes in South America [17,18]. We have previously identified limited genotypes in *T. gondii* isolates from humans, cats, pigs, sheep and birds in China [19-23], but there is little genetic information on *T. gondii* isolates from wild animals in China, especially on the Qinghai-Tibet Plateau, a region with a unique ecosystem. In the present article we describe the genetic characterization of *T. gondii* isolates from wild animals on the Qinghai-Tibet Plateau.

## Methods

### The investigated regions

The present study was conducted in Qinghai Province (31°-39°N, 88°-103°E), People’s Republic of China, which lies on the northeastern Tibetan Plateau, covering an area of 724,000 square kilometers, with an average altitude of 3,000 meters above sea level. The annual precipitation is below 400 mm and average annual temperature is between 5.7°C and 8.5°C, and the difference in daily temperature is large. The surveyed regions are of typical continental climate-altitude.

### Naturally infected wildlife

The animals examined in this study were captured by simple equipment named as ‘mousetrap’ by local governments to protect the grass of the Qinghai-Tibet Plateau. Brain tissue samples were collected from 48 Qinghai voles (*Microtus fuscus*), 101 Plateau pikas (*Ochotona curzoniae*) and 34 Tibetan ground-tits (*Pseudopodoces humilis*) from the Qinghai-Tibet Plateau. The tissues were kept in 70% ethanol directly after collection until further study.

### Extraction of genomic DNA and genetic characterization

Genomic DNA was extracted from approximately 100 mg of brain tissues by sodium dodecyl sulphate/proteinase K treatment, column-purified (Tiangen™, Beijing, China) and eluted into 50 μl H_2_O according to the manufacturer’s recommendations. A nested PCR targeting the *T. gondii* B1 gene was performed to detect possible infection with *T. gondii*[24]. DNA samples giving positive B1 amplification were then used for genetic characterization.

Genetic characterization of *T. gondii* isolates from these wild animals was carried out using the multilocus PCR-RFLP method [21,23,25,26]. In brief, the target DNA sequences were amplified by multiplex PCR using external primers for all 10 markers [25,26]. Six reference *T. gondii* strains were included as the positive controls including GT1, PTG, CTG, MAS, TgCgCa1 and TgCatBr5 (Table 1). The PCR reaction (25 μl) composed of 1× PCR buffer, 0.2 mM of each primer, 200 μM dNTPs, 2 mM MgCl_2_, 0.2 U of HotStart *Taq* DNA polymerase (TAKARA, Japan). The PCR amplification was performed using a thermal cycler (PTC 200, Bio-RAD). All samples were incubated at 95°C for 5 min to activate the DNA polymerase, then 30 cycles of PCR at 95°C for 30 s, 55°C for 60 s and 72°C for 90 s. Multiplex PCR-amplified products were diluted 1:1 in sterile, double-distilled water, and then used for nested PCR amplifications with internal primers for each marker, separately [25,26]. A similar program was used for the nested PCR. The nested PCR amplifications were carried out with the annealing temperature at 60°C for 60 s for all the markers except Apico, which was amplified at 55°C. The nested PCR products were digested with restriction enzymes for 1 h, and the temperature for each enzyme was used according to the instructions for each enzyme. The restriction fragments were resolved in 2.5% agarose gel, stained by the GoldenView™, and photographed using a gel documentation system (UVP GelDoc-ItTM Imaging System, Cambridge, U.K.).

**Table 1:** **Summary of genotyping of *Toxoplasma gondii *from Qinghai vole, Plateau pika and Tibetan ground-tit on the Qinghai-Tibet Plateau, China**

**Isolate ID**	**Host**	**Tissue**	**Location**	**SAG1**	**5’ + 3’ SAG2**	**Alternative SAG2**	**BTUB**	**GRA6**	**c22-8**	**c29-2**	**L358**	**PK1**	**Apico**	**Genotype**
GT1	Goat		United States	I	I	I	I	I	I	I	I	I	I	Reference, Type I, ToxoDB #10
PTG	Sheep		United States	II/III	II	II	II	II	II	II	II	II	II	Reference, Type II, ToxoDB #1
CTG	Cat		United States	II/III	III	III	III	III	III	III	III	III	III	Reference, Type III, ToxoDB #2
MAS	Human		France	u-1^*^	I	II	III	III	u-1^*^	I	I	III	I	Reference, ToxoDB #17
TgCgCa1	Cougar		Canada	I	I	II	III	II	II	u-1^*^	I	u-2^*^	I	Reference, ToxoDB #66
TgCatBr5	Cat		Brazil	I	III	III	II	III	I	I	I	u-1^*^	I	Reference, ToxoDB #19
TgOcDR40	Plateau pika	Brain	Qinghai, China	I	I	I	I	I	I	I	I	I	I	Type I, ToxoDB #10
TgOcDR1	Plateau pika	Brain	Qinghai, China	I	I	I	I	I	I	I	I	I	I	Type I, ToxoDB #10
TgMfDR39	Qinghai vole	Brain	Qinghai, China	I	I	I	I	I	I	I	I	I	I	Type I, ToxoDB #10
TgOcDR18	Plateau pika	Brain	Qinghai, China	I	I	I	I	I	I	I	I	Nd	I	Type I, ToxoDB #10
TgMfDR28	Qinghai vole	Brain	Qinghai, China	II/III	I	I	I	I	II	I	Nd	I	I	New genotype
TgPhDR1	Tibetan ground-tit	Brain	Qinghai, China	I	I	II	III	Nd	Nd	I	I	I	I	New genotype

* u-1 and u-2^*^represent unique RFLP genotypes, respectively.

Nd: represents no data.

## Results

Of 183 DNA samples, 11 were positive for the *T. gondii* B1 gene by PCR amplification, including 6 from Qinghai voles (6/48, 12.5%), 4 from Plateau pikas (4/101, 3.96%) and 1 from Tibetan ground-tit (1/34, 2.94%). Six DNA samples showed genotyping results, 3 from Plateau pikas, 2 from Qinghai voles, and 1 from Tibetan ground-tit (Table 1). Due to low DNA concentration, 5 of the 11 positive samples could not be genotyped completely or nearly completely, and was not used. Three genotypes were identified from the 11 positive samples, including Type I and two new genotypes (Table 1).

## Discussion

The Qinghai-Tibetan Plateau is a region that has a low pressure of oxygen and high ultraviolet radiation, where the altitude is high and the temperature is low. Little is known of the prevalence and genetic characterization of *T. gondii* in this extremely inhospitable high-altitude environment.

The plateau pikas are underground-dwelling relatives of rabbits, prefer to live in elevations of 3,100 to 5,000 m, mostly on the Tibetan Plateau. The plateau pikas are considered to be a reservoir of environmental spread of *T. gondii*, because they are prodigious breeders and serve as the favourite food of carnivores that live in the area, such as brown bears and wolves. *T. gondii* was first detected from plateau pika in 1994 using the indirect hemagglutination assay (IHA) [27]. Our present data first indicated that the three isolates from plateau pika on the Qinghai-Tibet Plateau belonged to Type I. This result is different from previous studies that showed the genotype ToxoDB PCR-RFLP genotype #9 was predominant in cats and other animals in southern, southwestern, and central parts of China [19-22,28]. This difference is probably because these animals live in a unique environment. Results of the present study indicate that Type I is the major clonal *T. gondii* genotype circulating in plateau pikas. To further identify the genetic diversity of *T. gondii* in plateau pikas, more samples from different geographical regions on the Qinghai-Tibet Plateau should be included.

Qinghai vole (*M. fuscus*) is a sub-species of small rodents on the Qinghai-Tibet Plateau. Vole populations can expand rapidly within a very short period of time, and many predators, such as martens, raccoons, owls, hawks, the red-tailed hawk, weasels, cats and dogs prey on voles. The seroprevalence of *T. gondii* infection in *Microtus fortis* was 29% in Hunan province [29] and 50.4% in Jilin province [30], China. Thus, voles can serve as a reservoir of environmental spread of *T. gondii*.

Little information is available about *M. fuscus* infection with *T. gondii*. The present study showed by PCR that a proportion (12.5%) of *M. fuscus* were positive for the B1 gene of *T. gondii*, and genetic analysis showed that one isolate is Type I (ToxoDB genotype #10), which may be a predominant genotype on the Qinghai-Tibet Plateau, and another isolate presents a new genotype (Table 1). In the present study, we also found that one Tibetan ground-tit was positive for the infection of *T. gondii*, and the isolate presents a new genotype (Table 1). A limitation of the present study is that the sizes of samples are not large, especially for Qinghai voles, only 48 samples were collected. To obtain more accurate information about the genetic diversity of *T. gondii* in these wild animals, more samples from different regions on the Qinghai-Tibet Plateau should be included. Although the role of wildlife in the transmission of *T. gondii* to humans and other animals is not fully understood [31], rodents and small animals are important intermediate hosts of *T. gondii* because they serve as a potential source of infection for some predators and may therefore contribute to the parasite’s spread.

The present work provides new genetic information about *T. gondii* infection in wildlife on the Qinghai-Tibet Plateau, China. *T. gondii* infection in wildlife is very important because people can become infected via eating undercooked meat.

## Conclusion

The present study genetically characterized *T. gondii* isolates from Qinghai vole, Plateau pika and Tibetan ground-tit on the Qinghai-Tibet Plateau, China, for the first time, and three *T. gondii* genotypes were determined (Type I and two new genotypes). These results provide new genetic information about *T. gondii* infection in wildlife on the Qinghai-Tibet Plateau, China, and have implications for our better understanding of the genetic diversity of *T. gondii.*

## Competing interests

The authors declare that they have no competing interests.

## Authors’ contributions

XQZ, SYH and WZJ conceived and designed the study and wrote and critically revised the manuscript. XXZ, ZZL and DHZ performed the experiments, analyzed the data, and drafted the manuscript. CS helped in the study design, study implementation, and manuscript revision. All authors read and approved the final manuscript
